# Nurses, the Oppressed Oppressors: A Qualitative Study

**DOI:** 10.5539/gjhs.v7n5p239

**Published:** 2015-03-16

**Authors:** Zahra Rooddehghan, Zohreh ParsaYekta, Alireza Nikbakht Nasrabadi

**Affiliations:** 1 School of Nursing and Midwifery, Tehran University of Medical Science, Iran, Tehran

**Keywords:** care, equity, nursing, oppression, Iran

## Abstract

Healthcare equity, defined as rightful and fair care provision, is a key objective in all health systems. Nurses commonly experience cases of equity/inequity when caring for patients. The present study was the first to explain nurses’ experience of equal care. A qualitative study sought to describe the experiences of 18 clinical nurses and nurse managers who were selected through purposive sampling. The inclusion criteria were the nurses’ familiarity with the subject of the study and willingness to participate. The data were collected through in-depth, unstructured, face-to-face interviews. The sampling continued up to data saturation. All the interviews were recorded and then transcribed word by word. The data were analyzed using thematic analysis. The major theme extracted in this study was the equation between submissiveness and oppression in nurses. It had two subthemes, namely the oppressed nurse and the oppressive nurse. The first subtheme comprised three categories including nurses’ occupational dissatisfaction, discrimination between nursing personnel, and favoring physicians over nurses. The second subtheme consisted of three categories, namely habit-oriented care provision, inappropriate care delegation, and care rationing while neglecting patient needs. When equal care provision was concerned, the participating nurses fluctuated between states of oppression and submissiveness. Hence, equal conditions for nurses are essential to equal care provision. In fact, fair behavior toward nurses would lead to equity nursing care provision and increase satisfaction with the healthcare system.

## 1. Introduction

Efforts to ensure healthcare equity are among the major objectives of health systems around the world. They are also the cornerstones of health programs implemented by various health systems and the World Health Organization (Rechel, Blackburn, Spencer, & Rechel, 2011). Healthcare equity, in its modern sense, is not only the allocation of medical resources to patients, but also providing life conditions to keep all people healthy (Aramesh, 2007).

Healthcare equity in nursing profession is no exception and should receive adequate attention (Iranian Nursing Ethics Code, 2011). Equity is a principle in healthcare ethics and an index of rightful and fair care provision (Rich & Butts, 2005).

During their professional life, nurses frequently face with decision-making situations where the feeling of equity or inequity dominates (Blais, Hayey, Kozier, & Erb, 2004). The equity principle necessitates similar behavior under equal conditions and different behaviors under unequal conditions (Chitty & Black, 2007). Therefore, healthcare equity is defined within three contexts: access to equal care for identical needs, equal use for identical needs, and equal quality of care for all people in need (Whitehead, 2000). Apparently, failure to provide equal healthcare services and supply the required resources for this purpose would increase social gap and result in negative social consequences (the Specialized Committee for Health and Bioscience, 2010).

Experiencing healthcare inequity can generally lead to public distrust of the health system, cause social resistance and conflicts, and evoke feelings of shock, inconvenience, anxiety, depression, disappointment, and betrayal. In other words, inequity can be stressful and thus harmful to public well-being and welfare (Johnstone, 2011).

On the other hand, proper and fair healthcare provision by nurses depends on the conditions of their workplace and their ability to provide high-quality care under such conditions. Moreover, nurses’ belief in their personal capabilities and self-consistency is critical to not only the quality of the provided care, but also their own feeling of liveliness in the workplace (Roberts, 1996). However, research has shown evidence of oppression and its consequences in nursing profession (Roberts, Demarco, & Griffin, 2009). Oppression is defined as a set of interrelated constraints and limitations which encourage obedience through practices to disdain, restrict, and shape individuals (Lee & Saeed, 2000; Duchscher & Myrick, 2008; Mooney & Nolan, 2006). Deutch (2006) described oppression as systematic, extensive, and recurring inequity. Cudd (2005) considered any unequal and unfair behavior toward others as oppression (Dong & Temple, 2011).

Oppression, a phenomenon experienced and reported by most nurses all over the world (Lee & Saeed, 2001), occurs when a dominant group develops a series of norms and regards outsiders as inferior. It is characterized by unfair behavior, ignoring others’ rights, and disrespecting their dignity. The presence and persistence of such behaviors would leave detrimental and irreversible effects on nurses and nursing profession, work organizations, and most importantly, patients and the quality of care they receive (Dinmohammad, Hushmand, Cheraghi, & Peyrovi, 2013).

According to Hulco (2009), since people tend to firmly resist being labeled as oppressive or oppressed, they may try to simultaneously fit in both groups, i.e. they may be oppressors from one perspective and oppressed from another (Dong & Temple, 2011). In the absence of comprehensive research to assess equity in healthcare in Iran, the dimensions of this crucial phenomenon in the country have remained unexplored. Hence, the present study aimed to explain the Iranian nurses’ experiences of equity in healthcare provision.

## 2. Methods

### 2.1 Design

This qualitative study employed thematic analysis to organize and analyze the data obtained from in-depth interviews (Smith & Sparkes, 2009).

### 2.2 Participant

The current study was performed in Iran. The study population comprised Iranian nurses of all nursing positions (from clinical nurses to nurse managers). Overall, 18 nurses (15 women and three men), aged between 29 to 61 years (age mean; 41/83), including 11 clinical nurses, three supervisors, one matron, one head nurse in public and private hospitals, and two members of the Nursing Council, were recruited using purposive sampling (years of work average; 17/38). The inclusion criteria were willingness to participate, having adequate experience in nursing care or nursing management, and being able to discuss the subject. The sampling continued until a point of data saturation was reached, i.e. until data redundancy occurred and the research questions were answered. Considering the subject of the study, diversity of the participants in terms of occupation, occupational position, education level, work experience, age, and sex was taken into account.

### 2.3 Data Collection

The data were collected using unstructured, in-depth, face-to-face interviews performed by the corresponding author. All interviews were conducted at the participants’ workplace and lasted for a mean duration of 45 minutes. The interviews were recorded using an audio recorder and immediately transcribed after each session.

The main questions were: “What are your experiences of equity in healthcare?”, “Which tasks do you regard as providing equal healthcare?”, “Which tasks do you regard as providing unequal healthcare?”, and “What do you believe can affect equity in healthcare?”. The interviews started with these general questions and were continued according to participants’ answers. The researchers listened to the interviews and reviewed the transcriptions several times.

### 2.4 Ethical Consideration

The study protocol was approved by Tehran University of Medical Sciences (Approval ID: 91D1302870). All the participants were asked to sign informed consent forms after explanation of the research objectives and their right to withdraw at any time of the study has been done. They were also provided with the researchers’ contact information for any question that they have. In addition, the interviews were only recorded with the nurses’ permission (they were informed that the audio files would be deleted after five years). In order to maintain the anonymity of the participants, numerical codes were used instead of names throughout the study (only the corresponding author was aware of the subjects’ names).

### 2.5 Data Analysis

Thematic analysis was used to analyze the collected data. As data collection and analysis were carried out simultaneously, each recorded interview was transcribed, read line by line, and encoded before the next one. The obtained codes were then compared with the previous ones and those with similar concepts were classified under the same category. In fact, data collection and analysis consisted of six steps including getting familiar with the data, extracting the initial codes, developing themes based on the extracted codes, reviewing the themes and comparing them with the data to ensure accuracy, defining and naming the themes, and preparing the final report (Braun & Clarke, 2006).

### 2.6 Rigor

In order to examine the accuracy and rigor of the collected data, Guba and Lincoln’s criteria (credibility, confirmability, dependability, and transferability) were evaluated. Two-year contact of the researcher with the data and the trust developed during the interviews increased data credibility. Some encoded interviews were randomly returned to the participants to confirm the accuracy of the data. A number of observers were also asked to review the codes. Moreover, two PhD candidates in nursing reviewed the data. The confirmability of the data was determined through recording the interviews and reflecting on the collected data by the researcher. Furthermore, using a sampling technique which provided maximum diversity in age, sex, work experience, and position contributed to data transferability. Finally, in order to enhance the transferability of the data, the exact procedures performed at various stages of the study were recorded (Streubert & Carpenter, 2003).

## 3. Results

The studied nurses fluctuated between states of oppression and submissiveness in the provision of equal care. Actually, the submissiveness of nurses in Iran’s health system led to their oppression. Therefore, when nurses were asked about equal healthcare, they indicated their conditions in the health system to be responsible for their inability to provide equal healthcare. This oppression/submissiveness equation was the most important theme extracted in this study. It included two main subthemes, namely the oppressed nurse and the oppressive nurse. Each subtheme comprised three categories (Table 1).

**Table 1:** Introduction of the subthemes and themes of the major category extracted in the present study

Theme	Sub theme
The oppressed nurse	Nurses’ occupational dissatisfaction
	Discrimination between nursing personnel
	Favoring physicians over nurses
The oppressive nurse	Habit-oriented care provision
	Inappropriate delegation of care services
	Care rationing while neglecting patient needs

### 3.1 The Oppressed Nurse

According to our findings, submissiveness refers to a series of conditions resulting from occupational dissatisfaction, discrimination between nursing personnel, and favoring physicians over nurses. Since nurses were not able to control such conditions, they had had to accept them.

#### 3.1.1 Nurses’ Occupational Dissatisfaction

The participants considered occupational dissatisfaction to be caused by disproportionate number of nurses and patients, inappropriate social status, lack of motivation, occupational burnout, and disproportionate workload and income. The nurses mostly introduced inadequate number of nursing personnel as the reason for excessive workload, job difficulty, and increased working hours in nurses. *“We are disastrously in shortage of human resources.”* stated a supervisor.

Moreover, the nurses explained that despite the shortage of personnel and high workload, they were not paid appropriately. *“This salary is low for this type of job”* mentioned the matron.

The participants also reported their inappropriate social status to reduce their motivation and lead to occupational burnout in the long time. *“Nurses are under a lot of occupational stress. They bear a heavy workload and that’s why they experience occupational burnout so early,”* an emergency nurse stated.

#### 3.1.2 Discrimination Between Nursing Personnel

The studied nurses described discrimination between nursing personnel as a factor leading to their submissiveness. Such discrimination manifested as different levels of workload assigned to different nurses, uneven distribution of nurses in terms of experience and skill in different shifts, lack of difference between efficient and inefficient personnel, and favoritism in allocation of nurses to various wards.

The participants perceived dissimilar levels of difficulty in tasks allocated to different nurses during a particular work shift. In other words, the unfavorable conditions of nurses in the health system were not equally distributed among the nursing personnel. This, in turn, resulted in further exploitation of the nurses. *“Considering the patients’ conditions, their allocation to nurses is very important. Sometimes, for instance, I am in charge of eight patients while my junior colleague has to take care of 10 patients. She may obviously not able to do that much.”* a nurse in the thoracic surgery ward believed.

Nurses and nurse managers emphasized that the staff shortage prevented the punishment of inefficient staff members. Meanwhile, failure to differentiate between efficient and inefficient nurses demotivated the first group and reduced their performance. *“Unfortunately, some personnel don’t work well, but we can’t expel them. Therefore, those working well are always complaining about inequity.”* explained a training supervisor in a public hospital.

Furthermore, nurses pointed out that their distribution in different wards was based on factors such as familiarity and favoritism rather than their expertise. *“Working in particular wards depends on relationships. For example, the acquaintance of X works in the CCU because it’s a stabilized, neat ward.”* a nurse with experience in nursing management expressed.

#### 3.1.3 Favoring Physicians Over Nurses

Another factor contributing to the submissiveness of nurses was favoring physicians over nurses. The significant difference in the income of physicians and nurses and greater support for physicians affected nurses’ perceived discrimination. In their attempts to highlight the existing discrimination, the studied nurses consistently compared their conditions with those of physicians. *“There is a pre-eminent group superior to not only the nursing system, but also the overall healthcare system. Physicians are not comparable to nurses. They differ in working hours, salary, challenges, and living standards. Physicians even receive unique facilities from the Ministry of Health!”* a clinical supervisor stated.

### 3.2 The Oppressive Nurse

We found the oppression practiced by the studied nurses to be a series of inevitable actions and behaviors in response to the oppression exerted on them. In other words, nurses transferred the oppression to others. This subtheme included three categories, namely habit-oriented care provision, inappropriate delegation of care services, and care rationing while neglecting patient needs.

#### 3.2.1 Habit-Oriented Care Provision

Due to perennial shortage of personnel, a particular healthcare pattern had turned into a care routine in nurses. Accordingly, care provision depended on the workload rather than patient needs. Nurses tended to pay little or no attention to communication with patients and sometimes underworked on the pretext of shortage of personnel. This working routine remained unchanged even when the number of nurses was equal to or greater than the number of patients. As a result, nurses became oppressors in the health system. *“Nurses have fallen into the habit. Imagine a ward with 30 patients and three nurses. Even if the number of nurses increase to seven, they would not work any harder.”* a male clinical supervisor indicated. Also the female clinical supervisor explained: *“A series of routines make nurses get used to some tasks. So, if the ward isn’t crowded, they do the same tasks. I see them make the same mistakes routinely even when the ward isn’t crowded.”*

#### 3.2.2 Inappropriate Delegation of Care Services

Due to the oppression faced by the nurses in the health system and their inability to change the existing conditions, nurses commonly delegate parts of their duties to the patients’ family members or other unprofessional individuals (e.g. workers of the ward) to make time for delivering all the necessary care services to the patients. Such practices might cause defects and errors in the care and threaten patient life. *“Sometimes, some colleagues don’t consider the patients’ right to get out of bed. They say it has to be done by health workers. I myself saw such a case in the general surgery ward where a colleague didn’t take it seriously. So, her patient got out of bed without knowing that his blood pressure could drop. The patient fainted and injured his head and had to have his head sutured. The patient had the right to know how he would feel by getting out of bed.”* a nurse in the thoracic surgery ward pointed out.

#### 3.2.3 Care rationing While Neglecting Patient Needs

Due to a shortage in facilities, the term *equity* is always accompanied by the term *rationing*. Meanwhile, oppressed nurses practice oppression in care rationing, i.e. they due not consider standards or patient needs in care rationing. They sometimes prioritize a patient’s needs based on factors such as the authorities’ request, their own idea, and the tasks they should complete at the end of the day. *“Sometimes, I’m really unfair to patients. Actually, when the physician orders too many tasks for a critically ill infant, I try to prioritize the tasks. For example, when an infant receives both milk and gavage-feeding and has to be injected with platelets or various drugs, I prefer to give him the drugs and follow up his platelets. I will then have to write that I’ve gavage-fed him with 1 cc. But unfortunately, no milk has been given to the infant because I can’t explain to the physician I haven’t give the drugs to the infant.”* a nurse in neonatal intensive care unit (NICU) reported.

## 4. Discussion

Based on our findings, oppressed nurses practiced behaviors which influenced customer-oriented care provision and made them act oppressively.

In a qualitative research, Kalisch (2006) introduced nine commonly neglected care practices as walking and moving the patients, timely feeding, patient instruction, discharge planning, emotional support, patient hygiene, recording the intake and excretion of patients, and monitoring. The researcher identified inadequate number of personnel, insufficient time for nursing tasks, poor use of available human resources, the “This is not my job” syndrome, inefficient management, habits, and denial to be responsible for such malpractices.

We also found low number of nurses, managerial problems, and lack of differentiation between efficient and inefficient personnel to turn nurses into oppressor. As a result, several were eliminated following care rationing and habit-oriented performance.

The nurses participating in the current study highlighted disproportionate numbers of nurses and patients as the main cause of various problems such as increased workload and working hours, unequal conditions, and low quality of care. Previous studies have also reported similar findings in this regard. Heede et al. (2009) conducted a study using administrative databases of all heart centers (including 58 critical care units and 75 general nursing divisions) in Belgium. They detected a significant correlation between higher number of nursing personnel in general wards and reduced postoperative mortality. However, increased number of nursing personnel in critical care units could not significantly decrease postoperative mortality. Hence, the researchers suggested the number of nursing personnel as a variable affecting both patient safety and the quality of the provided care. Rothberg, Abraham, Lindenauer, and Rose (2005) concluded that a patient/nurse ration of 8/1 was associated with minimum personnel cost and maximum mortality rate. Meanwhile, a ratio of 4/1 was accompanied by the highest personnel cost and the lowest mortality rate. They reported the existing ratios as 5-6/1 in the morning and afternoon shifts and 6-7/1 during the night shifts. Nevertheless, the ratios are much larger in Iran.

Our finding also reflected discrimination between nursing personnel as another dimension of oppression which influenced the nurses’ customer-oriented behavior. In a study on 478 male and female nurses working in hospitals affiliated to Isfahan University of Medical Sciences (Isfahan, Iran), Golparvar and Nadi (2009) showed customer-oriented behaviors in nurses to have significant positive correlations with perceived distributional equity (nurses’ perception of fair outcomes) and perceived procedural equity (nurses’ perception of equity in decision-making practices). In other words, when nurses believed in the fairness of the procedures and methods involved in decision-making about their conditions, they developed greater trust in the fairness of the outcomes and thus treated the patients with more favorable behaviors.

Our participants considered the discrimination between physicians and nurses as another factor contributing to oppression. Despite their academic education and substantial skills, nurses in many other parts of the world have also been shown to receive lower salary than their peer physicians. Therefore, nurses are oppressed in this regard (Lee & Saeed, 2001; Hagbaghery, Salsali, & Ahmadi, 2004).

In addition, the nurses in the present research had to ration their duties due to their occupational limitations. Recent studies on nursing care rationing have revealed serious threats to the quality of care and patient safety following the rationing of time and care practices by nurses. Correlations have been discovered between care rationing and not only decreased patient satisfaction, but also increased incidence of falls, nosocomial infections, bedsores, and mortality among the patients. Experimental evidences have clearly shown that the quality of care and safety have been strongly damaged in the contemporary nursing (Papastavrou, 2013). In fact, nurses have to ration and prioritize the caring practices due to the limited resources and facilities. However, oppressed nurses generally ration care as a result of their occupational status rather than the fulfillment of patient needs. According to Papastavrou (2012), the philosophy of care, which refers to the nurses’ beliefs, values, and ideals, is influenced by economic and time limitations. For instance, nurses who have limited access to healthcare resources may feel that comprehensive human care practices are not realistic. They may thus adjust their personal standards to match the existing limitations. Scheunemann (2011) defined rationing as critical structural considerations in the development of a fair, appropriate healthcare system. Consequently, levels at which healthcare practices are rationed and transparency of this ethical rationing should be chosen consciously using logical principles and fair methods. However, in the present study, instead of following particular principles, the nurses performed rationing based on their own conditions.

Inappropriate care delegation was another category extracted in the current research. Nurses in the 21^st^ century need to be able to delegate care services and to supervise. The delegation of care provides registered nurses (RN) with more time to caring for, support, and instruct the patients. Although various guidelines have been introduced in this regard (AACN, 2004), there are no specific instructions for delegation of care in Iran’s nursing care system. On the other hand, due to the limited number of practical nurses, they are sometimes obliged to delegate care practices to unprofessional people such as patient families. However, there is no appropriate supervision on this delegation of care due to the shortage of personnel and high workload. Such conditions disturb the appropriate delegation of nursing care and make nurses behave oppressively toward the patients. Similarly, Gravlin and Bittner (2010) reported the knowledge, skills, and attitudes of practical nurses and workload as factors affecting successful delegation of nursing care.

Another dimension of the nurses’ oppressive behaviors in the present study was habit-oriented care provision while neglecting patient needs and professional standards. Kalisch (2006) indicated that in an attempt to adapt with eliminated care services, nurses adjusted their care practices based on the instructions of physicians. Finally, these behaviors resulted in basic routine nursing care which never fully flourished.

## 5. Conclusion

According to our findings, nurses fluctuated between states of oppression and submissiveness in provision of equal care. Therefore, equal conditions for nurses are essential to equal care provision. Since the exploitation of nurses can eventually cause irreversible effects on the quality of the provided care, patient safety and satisfaction closely correlate with the care provision atmosphere and nurses’ satisfaction with their occupation and social status.
